# Extracorporeal shockwave therapy combined with multiple drilling and intramedullary drug injection for treating early-stage Femur Head Necrosis: Protocol for a randomized controlled trial

**DOI:** 10.1097/MD.0000000000022598

**Published:** 2020-10-02

**Authors:** Qianchun Li, Rigao Chen, Yang Yu, Xinling Wang, Xueya Feng, Leiming Jiang, Botao Chen, Peng Xin, Tong Li, Yin Shi, Qiang Jian, Zhongchao Jiang, Xiaohong Fan

**Affiliations:** a School of Clinical Medicine, Chengdu University of Traditional Chinese Medicine; b Department of Orthopedics, Hospital of Chengdu University of Traditional Chinese Medicine, Chengdu; c Department of Anorectal Surgery, Nanchong Central Hospital, Nanchong; d Department of Intensive Care Unit, JianGe County Hospital of Traditional Chinese Medicine, Guangyuan, Sichuan, China.

**Keywords:** extracorporeal shockwave therapy, femur head necrosis, intramedullary drug injection, multiple drilling

## Abstract

**Background:**

Early diagnosis and treatment of the osteonecrosis of the femoral head (ONFH), a refractory disease, is imperative to prevent femoral head collapse; however, the existing solutions remain controversial. This study assessed the safety and efficacy of extracorporeal shock wave therapy (ESWT) combined with multiple drilling and intramedullary drug injection, a novel cocktail therapy, as a randomized controlled trial (RCT) model to postulate an alternative therapy for patients with early-stage ONFH.

**Methods:**

Femoral head necrosis patients aged 20 to 60 years with stage ARCO I-II were recruited. One hundred twenty eligible participants were randomized into four groups in a 1:1:1:1 ratio: extracorporeal shock wave therapy combined with multiple drilling and intramedullary drug injection (group EMI), extracorporeal shock wave therapy (group E), multiple drilling combined with intramedullary drug injection (group MI), and multiple drilling (“positive” control group; group M). The primary outcomes included effective rate, subchondral collapse rate of the femoral head, lesion size, and grade of bone marrow edema. Secondary outcomes included the Harris Hip Score and the visual analog scale. All outcomes were measured at the screening visit (baseline) and at the planned time intervals during treatment and follow-up, and the efficacy was statistically analyzed according to the intention-to-treat sub-populations and per-protocol sub-populations.

**Objectives:**

To examine the clinical efficacy of ESWT combined with multiple drilling and intramedullary drug injection to provide a safe and more effective method for treating early-stage ONFH.

**Trial registration number:**

ChiCTR1900020888; Pre-results.

## 1 Introduction

The characteristics of osteonecrosis of the femoral head (ONFH), a treatment-resistant disease, include degradation of the osteocytes as well as the bone marrow, causing severe hip pain and arthritis, with the bilateral incidence in ∼75% of the cases.[^1^,^2^,^3^] In the United States, ∼15,000 to 20,000 cases of ONFH are diagnosed annually.[^4^,^5^] In China, ∼8.12 million ONFH patients are aged ≥15 years and there is an annual incidence rate of 150,000 to 200,000.[^6^,^7^] Due to the lack of efficient therapies, both symptomatic (85%) and asymptomatic (67%) patients suffer from the subchondral femoral head collapse, which requires total hip replacement/arthroplasty (THA).^[8]^ Glucocorticoid or alcohol-related ONFH manifests not only as a femoral head lesion but also spreads and impairs the bones throughout the body. Such patients with THA have a higher incidence of complications, and the long-term effects are not ideal.^[9]^ Young patients might require one or more revision surgeries in their lifetime, accompanied by tremendous pain as well as financial burden.^[6]^

Thus, it is necessary to find alternative strategies for early diagnosis as well as simple and effective treatments to block the progress of osteonecrosis to avoid femoral head collapse and THA. Early diagnosis presents several challenges. Early identification and treatment of patients with acute pain in the hip and groin area could be helpful in improving the diagnosis.[^10^,^11^] In recent years, extensive research to investigate hip preservation methods related to ONFH has been done; however, there is still limited evidence on this disease.[^3^,^4^]

Core decompression (CD), often used in conjunction with concurrent nonvascularized bone grafting, is a common method for treating the pre-collapse.^[5]^ Based on CD, multiple drilling decompression was developed, which can achieve “decompression,” while avoiding the disadvantages of iatrogenic collapse caused by the loss of mechanical support of the femoral head due to the large diameter of decompression.^[12]^ For ARCO stage I-II patients, studies have found no difference in efficacy between core decompression with or without graft and percutaneous multiple drilling. Percutaneous drilling, which can target the lesion relatively easily, can effectively lessen the intramedullary pressure and enhance microcirculation in the subchondral region; thus, and promoting angiogenesis in the necrotic cavity.[^1^,^13^] This technique is accessible and minimally invasive, and has been used extensively with encouraging results.[^14^,^15^]

Since 2001, clinicians have been interested in exploring the use of extracorporeal shock wave therapy (ESWT), a biophysical therapy for treating early-stage femoral head necrosis.^[16]^ Although there is limited information on the mechanism of action of ESWT, it is hypothesized to be linked to elevated angiogenic growth factor levels to stimulate neovascularization ingrowth, which would, in turn, cause cellular proliferation and osteogenesis.^[17]^ Previous studies have revealed that ESWT is more efficacious than CD and bone grafting for early-stage ONFH.^[18]^ Based on current evidence, ESWT has a definite effect on reducing bone marrow edema, alleviating pain, and improving function early-stage ONFH patients; however, it is difficult to reverse necrotic bone, and there is insufficient evidence to improve the prognosis of ONFH and reduce the rate of THA. Moreover, when used in combination with other non-surgical conventional therapies, no significant difference was observed in the efficacy of ESWT.^[17]^

This study studied another treatment that has not been reported internationally: intramedullary drug injection. This method was the first reported in 2000 by Professor Zhongchao Jiang in China, which has been used in clinical practice for a long time with good clinical efficacy (48.8–80%), but most of them are retrospective studies with insufficient evidence.

Despite extensive research on the treatments for the early-stage femoral head necrosis, such as CD with/without graft, bone-marrow implantation, bone grafting with/without vascularization, growth-factor-based treatment, mesenchymal stem cells, and proximal femoral osteotomies, there is still little evidence of treatment in stage I patients, and the optimal joint protection method is still unknown.^[11]^ The clinical efficacy of a monotherapy may be very limited. Based on previous research and clinical practice, we hypothesized:

1. The new cocktail of extracorporeal shock wave therapy + multiple drilling + intramedullary drug injection could achieve better clinical efficacy,
2. The clinical effect of ESWT alone would be no worse than that of multiple drilling, and
3. the clinical efficacy of intramedullary drug injection would be explored.

Thus, here an RCT protocol was formulated to verify this hypothesis.

## 2 Methods and analysis

### 2.1 Objective

To examine the clinical efficacy of ESWT combined with multiple drilling and intramedullary drug injection to provide a safe and more effective method for treating early-stage ONFH.

### 2.2 Study design

**Figure 1:**
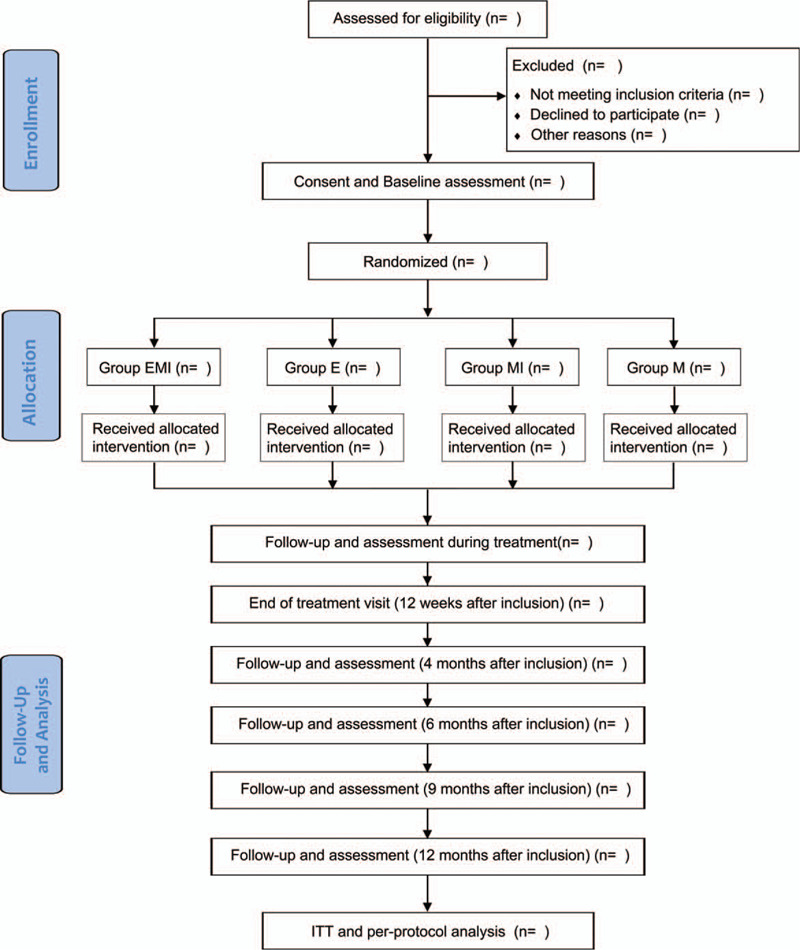
Flowchart of the trial design. E = extracorporeal shockwave therapy, EMI = ESWT + multiple drilling + intramedullary drug injection, ITT = intention-to-treat, M = multiple drilling, MI = multiple drilling + intramedullary drug injection.

This study is a prospective, single-center RCT, which was designed in accordance with the principles of the Declaration of Helsinki. All participants would be provided in-depth information on the protocol, risks, and right to withdrawal from the research. A signed informed consent would be procured from all participants. The protocol adheres to the SPIRIT 2013 Statement.[^19^,^20^] This trial has been registered at the Chinese Clinical Trial Register: ChiCTR 1900020888. Figure 1 provides the flowchart of the trial design. Protocol version: Z2.2, Version date: 20200531.

### 2.3 Recruitment strategies

Posters for recruitment would be posted in the outpatient and orthopedic inpatient departments of the Hospital of Chendu University of Traditional Chinese Medicine. Other methods of recruitment would include local print media, social media, flyers, and word of mouth. Formal recruitment for the study began in June 2020 and would continue until June 2021. Patients who would consent to participate would be examined and diagnosed by the physician (FXH, JZC).

### 2.4 Inclusion criteria

1. Patients between the ages of 20 and 60 years.
2. Patients with a clinical diagnosis of ONFH,^[21]^ staged as ARCO I-II.^[22]^
3. Patients who would voluntarily participate in the trial and agree to sign the relevant written consent.

### 2.5 Exclusion criteria

1. Patients with a history of hip fracture or surgery
2. Patients who consumed hormones or alcohol within the last 1 month.
3. Patients undergoing any other treatment.
4. Patients who were pregnant or lactating.
5. Patients who were allergic to relevant drugs used in the course of this test.
6. Patients with other hip diseases or serious medical diseases.

### 2.6 Randomization, allocation, and blinding

The randomization sequence would be generated in advance by the clinical supervisor (WXL) using STATA (v15.1, StataCorp LLC, USA), and would be sealed in opaque envelopes. After the baseline testing, the researchers (LQC, CBT) would open the envelopes by serial number in the presence of the clinical supervisor (WXL). It was difficult to implement the blinding since the treatment methods could be known by the subjects and the doctors. The efficacy and safety evaluation would be performed by blinded third-party data analysts.

### 2.7 Therapies

#### 2.7.1 Extracorporeal shock wave therapy

Here, we would use the Orthopedic ESWT Device (EMS Swiss DolorClast, FT-174). The patient would be placed in the supine position with adduction and internal rotation of the affected limb. The anterior and the lateral part of the hip would be marked at 3 to 5 points using an X-ray or ultrasound depending on the lesion size. Lidocaine gel would be applied to the skin at those points. During treatment, attention would be paid to avoid shock wave damage to the femoral arteries and nerves. The study would include two courses of treatment; each course would include weekly treatments for 4 weeks, and each time 3000 to 5000 pulses (1000 pulses/spot at an energy flux density of 0.20–0.30 mJ/mm^2^) would be administered, followed by rest for 1 month, after which, the next course would be initiated.[^23^,^24^,^25^]

#### 2.7.2 Multiple drilling

Based on the X-ray, CT, or MRI scans, the direction, position, depth, and the number of drilling holes (generally 2–3) would be designed in advance. The patients would be in the supine position, with the affected hip raised 5 to 10 cm with a 15° internal rotation. The insertion area would be draped aseptically, followed by the administration of local anesthesia. A 2.5 mm Kirschner wire would be inserted percutaneously, 3 to 5 cm distal to the greater trochanter, and would be fluoroscopically guided towards the necrotic area until 0.5 cm beneath the subchondral bone.^[26]^ It usually takes 3 to 4 different tunnel bores.^[11]^ When finished, the pin would be removed, followed by a sterile dressing. This treatment would be done only once.

#### 2.7.3 Intramedullary drug injection

The patient would be placed in the supine position, with the affected hip raised 5 to 10 cm with a 15° internal rotation to expose the greater trochanter of the femur. After skin preparation and draping, anesthesia along with 10 ml of 5% lidocaine would be administered to infiltrate from skin to the periost, layer by layer. The lateral greater trochanter of the affected hip was designated as the insertion point, and an 18-gauge puncture needle was penetrated into the cancellous bone area of the femur neck fundus, followed by extraction of the needle core along with 5 to 10 mL of medullary blood using a 10 mL syringe. Then, the compound osteopeptide injection (5 mL) and Chuanxiong Danshizine injection (5 mL) were slowly injected (with no apparent local pain). After the injection, the needle core was slowly introduced, the puncture needle was pulled out, and a sterile gauze was applied for compression dressing; thus, completing the operational procedure. This treatment would be administered weekly for a period of 4 weeks, followed by a 1-month resting period, and repeated. This study treatment would include two courses.

### 2.8 Interventions

#### 2.8.1 Group EMI (ESWT + multiple drilling + intramedullary drug injection)

In this group, the first course of treatment would include ESWT, followed by Multiple drilling and intramedullary drug injection. Then, for the next 3 weeks, they would receive ESWT and intramedullary drug injection on a weekly basis. After a 1-month resting period, the patients would receive weekly ESWT and intramedullary drug injection for 4 consecutive weeks.

#### 2.8.2 Group E (extracorporeal shockwave therapy)

The patients in this group would receive two courses of ESWT.

#### 2.8.3 Group MI (multiple drilling + intramedullary drug injection)

The patients in this group would receive multiple drilling and an intramedullary drug injection as the first treatment. Then, they would receive an intramedullary drug injection on a weekly basis for 3 weeks. After a 1-month rest period, the patients would receive weekly intramedullary drug injection for 4 consecutive weeks.

#### 2.8.4 Group M (multiple drilling)

The affected hip would undergo the multiple drilling processes once to decompress, and the patients would be followed-up.

All patients participating in the trial would receive rehabilitation guidance and training.

### 2.9 Outcome measurements

Table 1 lists the data collection times.

**Table 1:**
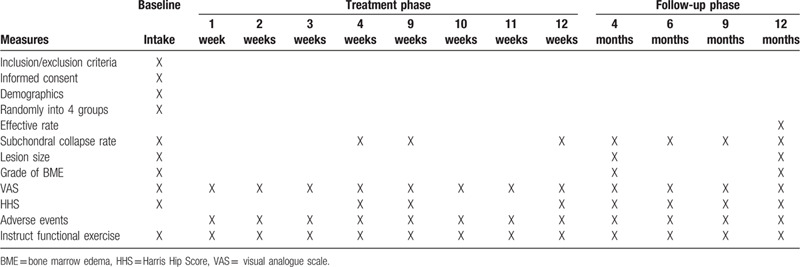
Time of data collection.

#### 2.9.1 Primary outcome

##### 2.9.1.1 Effective rate

The effectiveness would be evaluated at the last follow-up visit. The intervention would be considered effective if the necrotic area of the femoral head does not increase, and the ARCO stage does not progress. Effective rate = (Treatment effective hips/total number of hips participating in follow-up) × 100%.

##### 2.9.1.2 Subchondral collapse rate of the femoral head

The subchondral femoral head collapse would be evaluated by plain Radiography or CT scans.

##### 2.9.1.3 Lesion size

The lesion size would be determined by 3-D MRI measurements.^[27]^ The entire femoral head and the necrotic area would be manually outlined in each coronal image, and the area of the outline would be calculated automatically by PACS (Picture and Archiving Communications System, Chetu Inc, USA). For each hip, lesion size = X/Y × 100%, where X = the sum of the necrotic area on each coronal MRI section, and Y = the sum of the entire femoral head area on each coronal MRI section.

##### 2.9.1.4 Grade of bone marrow edema (BME)

The patients would undergo an MRI scan before inclusion as well as 4 and 12 months after inclusion to evaluate the BME of the femoral head. BME would be classified as follows: grade 0 (no BME), grade 1 (perinecrotic BME), grade 2 (BME extended into the femoral head), grade 3 (BME extended into the neck of the femur), and grade 4 (BME extended into the intertrochanteric region).^[28]^

#### 2.9.2 Secondary outcome

##### 2.9.2.1 Visual analogue scale

VAS would be used to evaluate the pain degree of the affected hip.^[29]^

##### 2.9.2.2 Harris hip score (HHS)

HHS would be used to evaluate the hip function of the affected side.^[30]^

#### 2.9.3 Baseline demographics

The following baseline demographic parameters would be recorded: age, gender, course of the disease, ARCO stage, history of smoking, alcohol consumption, glucocorticoid use, and concomitant disease.

### 2.10 Sample size

The primary outcome would be the effective rate (the percentage of no clinical-stage progression and no increase in necrosis area). Previous studies have revealed the effective rate of multiple drilling to be 61.5%,^[31]^ and the effective rate of ESWT to be 78.45%.^[25]^ Based on these results and our pilot study, we conservatively estimated the effective rate of the MEI group (the treatment group) to be 95.0%. The sample size of group MEI (the treatment group; 20) and group E (the control group; 20) would achieve 80.232% power to detect a difference in the effective rate between the two groups. Statistical analysis would be done using a two-sided *Z*-Test with unpooled variance. The significance level of the test would be .05 (PASS v15.0.5, Power Analysis and Sample Size). Due to the limited sample size, this study would conduct a non-inferiority study of ESWT compared with multiple drilling. Sample sizes of the group E (24) and group M (24) would achieve 81.098% power to detect a non-inferiority margin difference between the group proportions of −0.20. Statistical analysis would be done using the one-sided *Z* test (unpooled). The significance level of the test would be .025. Additionally, we also designed group MI (multiple drilling + intramedullary drug injection) to observe the efficacy of this method and to explore whether the addition of the intramedullary drug injection could further increase the efficacy compared with the control group (group M); compared with group EMI, based on group MI, whether ESWT could further increase the efficacy. Estimating a dropout rate of 20%, we planned to enroll a total of 120 patients, with 30 patients per group.

### 2.11 Data collection, management, and analysis

#### 2.11.1 Data collection

Baseline data would be collected before grouping; outcome and other trial data would be collected as planned during treatment and follow-up **(** Table 1). The efficacy and safety evaluation would be performed by a third-party blinded analysts.

#### 2.11.2 Data management

The researchers involved in the trial would be trained in good clinical practice. The original data would be recorded correctly on the case report form (CRF) on a timely basis, which would be reviewed and signed by the inspector and handed over to the data managers. Investigators would also log into the Clinical Trial Management Public Platform (www.medresman.org) to enter the data, and data managers would review the electronic data for consistency with the data on the CRF. During this period, if any problem occurs, the inspector shall be notified on time, and the investigator would be questioned. All questions/answers between them would be exchanged in the form of question sheets, which would be preserved for future reference. The paper and the electronic version of the CRF would be kept in the Hospital of Chengdu University of Traditional Chinese Medicine for 10 years, with restricted access.

#### 2.11.3 Statistical methods

Continuous data with a normal distribution pattern would be presented as mean ± standard deviation (SD) and analyzed using the one-way ANOVA, and LSD-*t*-tests would be used for multiple comparisons. Data with a non-normal distribution pattern would be presented as median (interquartile range) and analyzed using the Kruskal–Wallis *H* test; Nemeyi tests would be applied for multiple comparisons. For the ordinal data, we would use the Kruskal–Wallis *H* test to assess the difference between the four groups; and Nemeyi tests would be used for multiple comparisons. Categorical variables would be expressed as frequencies and percentages and analyzed with the chi-square test or the Fisher's exact test. We would use the Cox's proportional hazard model to examine the survival status of the subchondral femoral head collapse between the four groups. Repeated measurement ANOVA would be used to analyze the repeated measurement data, such as VAS scores and HHS. Statistical analyses would be done according to the intention-to-treat principle on SPSS 22.0 Windows (IBM Corp, USA) with two-tailed tests wherever appropriate, and *P* < .05 would be regarded as statistically significant.

### 2.12 Monitoring

#### 2.12.1 Data monitoring and auditing

The Operational Guidelines for the Establishment and Functioning of Data and Safety Monitoring Board by the WHO would be used as the reference to establish the Data and Safety Monitoring Board. The committee would be an independent advisory group and would ensure the quality of research as well as protect patient rights and health. Every three months, the Data and Safety Monitoring Board would conduct a surveillance inspection and appraisal. Additionally, the ethics committee would conduct a follow-up review every 12 months.

The project leader could suspend the trial and report to the results to the ethics committee and project sponsor if serious safety problems are found during the trial or the treatment effect of the test group is found to be too poor, even invalid with no clinical value, or other reasons.

#### 2.12.2 Harms

All adverse events would be routinely reported and documented during the treatment and follow-up. An adverse event is defined as any undesirable experience experienced by the study participant, which may/may not be directly related to the intervention. All adverse reactions will be handled appropriately.

### 2.13 Patient and public involvement

Neither the patients nor the public would have any involvement in the design/conduct of this study, and in the outcome measures.

### 2.14 Ethics and dissemination

The Medical Ethics Committee of Hospital of Chengdu University of Traditional Chinese Medicine provided ethics approval for this clinical trial (ID: 2019KL-042). If the protocol needs to be amended, approval from the ethics committee is required before implementation. The results of this study would be published in peer-reviewed journals and presented at international conferences. All forms of reports will be kept confidential for participants.

## 3 Discussion

Currently, there is limited evidence regarding the pathological mechanism of ONFH. It is known that venous stasis or damage or interruption of the arterial blood supply causes the apoptosis of bone cells and bone formation-related cells, resulting in sparse trabecular bone and micro-fractures. This induces the initiation of the self-healing process, while simultaneously causing immune damage and the formation of osteosclerosis band. The osteosclerosis band hinders the growth of new capillaries, which accelerates the progression of necrosis in the bone tissue and causes the femoral head collapse and secondary osteoarthritis in the late stage.^[21]^ The current techniques used for the early diagnosis and treatment of ONFH to avoid the femoral head collapse are controversial. One of the common techniques used pre-collapse includes CD with bone graft. However, there exists a risk of overtreatment for ARCO I-II patients. Recent studies on the use of CD combined with mesenchymal stem cells/bone-marrow implantation/growth-factor-based treatment are expected to achieve better results.[^1^,^2^,^32^] However, some studies have shown that due to the significantly reduced quality and quantity of mesenchymal stem cells in patients with ONFH with a history of corticosteroids or alcoholism, these cells are less likely to differentiate into osteocytes.[^33^,^34^,^35^] It is concerned that precursor cell implantation and growth-factor-based treatment, such as bone morphogenetic protein (BMP), may also cause serious complications. Thus, further studies are required to evaluate the efficacy of these therapies.

According to the pathological mechanism of ONFH, combined with previous research and clinical practice, we hypothesized that the new cocktail therapy of ESWT + multiple drilling + intramedullary drug injection could achieve better clinical efficacy in the treatment of early-stage ONFH. First, multiple drilling (2.5 mm) could not only realize “decompression,” but could also break through the osteosclerosis band barrier, providing a channel for the drainage of necrotic liquefied tissue in the femoral head as well as the growth of new capillaries.[^14^,^36^,^37^] Second, ESWT could promote the repair and reconstruction of the tissue,^[38]^ dilate and regenerate blood vessels,^[39]^ and also have an analgesic,^[40]^ anti-inflammatory,^[41]^ and high-density tissue splitting decomposition effect.^[42]^ Finally, intramedullary drug injection, a unique treatment that has been reported in several literature reports in China, is beneficial in achieving and maintaining effective local blood drug concentration. Compound Ossotide has the pharmacological effects of regulating calcium and phosphorus metabolism, stimulating osteoblasts proliferation, regulating bone metabolism, promoting new bone formation, and increasing bone calcium deposition. Danshen ligustrazine, an extract of Traditional Chinese medicine, is known to inhibit platelet aggregation, promotes antithrombotic function, and dilates arteriole to improve microcirculation. The combined action of these two drugs promotes the reconstruction of local blood circulation and bone tissue repair and reconstruction. Although the drug is injected into the medullary cavity at the base of the femoral neck, it acts on the lesion through the microcirculation of the proximal femur, and the decompression of the drill hole establishes the channel for the drug to reach the necrotic area. The combined application of these three methods is expected to achieve excellent clinical efficacy.

If this hypothesis is verified, our findings will provide a minimally invasive, safe, accessible, and effective treatment for patients with early-stage ONFH and provide evidence-based medical recommendations for the clinical promotion of this treatment.

## Author contributions

**Conceptualization:** QianChun Li, Rigao Chen, Yang Yu, Xinling Wang, Zhongchao Jiang, Xiaohong Fan.

**Data curation:** QianChun Li, Rigao Chen, Yang Yu, Xueya Feng, Leiming Jiang, Botao Chen, Peng Xin, Tong Li, Yin Shi, Qiang Jian, Zhongchao Jiang, Xiaohong Fan.

**Formal analysis:** QianChun Li, Rigao Chen, Yang Yu, Xueya Feng, Zhongchao Jiang, Xiaohong Fan.

**Funding acquisition:** Xiaohong Fan.

**Investigation:** QianChun Li, Rigao Chen, Yang Yu, Xinling Wang, Botao Chen, Peng Xin, Tong Li, Yin Shi, Qiang Jian, Zhongchao Jiang, Xiaohong Fan.

**Methodology:** QianChun Li, Rigao Chen, Yang Yu, Xinling Wang, Zhongchao Jiang, Xiaohong Fan.

**Project administration:** Rigao Chen, Xinling Wang, Zhongchao Jiang, Xiaohong Fan.

**Resources:** Zhongchao Jiang, Xiaohong Fan.

**Software:** QianChun Li, Xueya Feng.

**Supervision:** Rigao Chen, Xinling Wang, Xiaohong Fan.

**Validation:** QianChun Li, Rigao Chen, Yang Yu, Zhongchao Jiang, Xiaohong Fan.

**Visualization:** QianChun Li.

**Writing – original draft:** QianChun Li, Xueya Feng, Botao Chen, Tong Li, Yin Shi, Qiang Jian.

**Writing – review & editing:** Rigao Chen, Yang Yu, Xinling Wang, Leiming Jiang, Peng Xin, Zhongchao Jiang, Xiaohong Fan.
